# Short tandem gene duplications as potential agents of genetic memory

**DOI:** 10.1128/mbio.02011-25

**Published:** 2025-10-16

**Authors:** Samuel Lee, Matthew Radey, Pradeep Singh, Colin Manoil

**Affiliations:** 1 Department of Genome Sciences, University of Washington Seattle 7284, Seattle, Washington, USA; 2 Department of Microbiology, University of Washington Seattle 7284, Seattle, Washington, USA; Center for Microbial Dynamics & Infection, Atlanta, Georgia, USA

**Keywords:** heteroresistance, *aeruginosa*, intrinsic, aminoglycoside, amplification, antibiotic

## Abstract

Tandem gene duplications are spontaneous mutations that occur frequently but whose significance is often overlooked because they are genetically unstable and can be difficult to detect by sequencing. Here, we studied the spontaneous loss, amplification, and phenotypic effects of engineered duplications giving rise to antibiotic resistance in *Pseudomonas aeruginosa*. The duplications, ranging from 2.6 to 497 kb in length, were constructed using phage lambda *red* recombination of PCR products and carry resistance markers at their novel junctions. The genetic behaviors of the duplications depended on segment length: long duplications were readily lost during growth without selection and showed limited amplification potential, whereas short duplications were lost slowly and could amplify to high segment copy numbers. The shortest duplications analyzed (2.6 and 3.3 kb) persisted during extended growth without selection and are predicted to be stable for thousands of generations. Following growth without selection, the duplications retained their ability to amplify and generate extreme antibiotic resistance. The remarkable stability of short duplications combined with their capacity to amplify suggests that they can regenerate gene amplifications which were previously selected and then lost, a novel form of genetic memory. This feature could be particularly significant in adaptation to intermittent stresses, such as during cycled antibiotic treatment of chronic infection.

**IMPORTANCE:**

Unstable mutations, such as tandem gene duplications, contribute significantly to the evolution of antibiotic resistance and other traits, but their importance is often underestimated. In this study, we characterized duplication genetic behavior in the ESKAPE pathogen *Pseudomonas aeruginosa*. The work helps define the genetic behavior of duplications in *P. aeruginosa* and provides a framework for evaluating their potential roles in the organism’s well-known capacity to adapt to new environments.

## INTRODUCTION

Tandem gene duplications and higher amplifications occur frequently and lead to overexpression of amplified genes, but they are often overlooked because they are unstable and difficult to detect using standard genome sequence short read assembly methods (1–3). Duplications with sizes ranging from a few kilobases to several megabases have been reported and can occur over the entire genome (1, 4). Previous work in *Salmonella* suggests that ~10% of bacteria in growing cultures contain gene duplications somewhere in the genome, and for any individual site, duplications exist at frequencies of ~10^–2^ to 10^–5^ per cell (1).

The behavior of gene duplications has been characterized in model bacteria and has been lucidly reviewed (1, 5, 6). A hallmark of tandem duplications is recombinational instability in the absence of selection, with loss rates as high as 0.06 per cell per generation (4). Loss can be accelerated by fitness costs of carrying duplications, particularly if they are long. Amplification of duplicated regions by recombination also occurs readily, leading to multiple tandem copies of the duplicated segments and increased expression of encoded proteins. The genetic instability of gene amplifications makes them challenging to detect and analyze.

Tandem gene amplification frequently causes genetically unstable antibiotic resistance and can underlie heteroresistance, in which a clonal population of sensitive bacteria contains a high frequency of resistant members (7, 8). When screened for using appropriate methods, heteroresistance is seen frequently and in multiple species, including *P. aeruginosa* (2, 9–14). Gene amplification-mediated resistance results from the increased expression of antibiotic-inactivating enzymes, efflux pumps, or antibiotic targets encoded by amplified genes (3, 15, 16). Other expression-limited phenotypes, such as the growth rate of *P. aeruginosa* on adenosine, can also be enhanced by gene duplication and amplification (17, 18).

Because of the prevalence and importance of tandem gene duplications, a better understanding is needed of the factors affecting their stability and copy number change, and the effects of genome context. Here, we sought to help define the features governing these behaviors in *P. aeruginosa* by analyzing a defined set of duplications of different lengths constructed by a targeted procedure analogous to one developed in *Acinetobacter baylyi* (18). We found that long duplications were much more unstable than short duplications. The remarkable stability of very short duplications, combined with their ability to amplify by recombination, should allow lineages that carry them to “remember” and regenerate past adaptive amplifications selected in encounters with antibiotics and other stressful conditions but then lost during growth without selection.

## RESULTS

### Rationale

The overall objectives of this study were to examine the loss and amplification behaviors of gene duplications that varied in size and to evaluate how the behaviors affected phenotypes dependent on gene dosage. To do this, we developed a procedure to construct defined duplications in *P. aeruginosa* and examined the loss and higher-level amplification of a set of such duplications affecting the same genomic region. The approach made it possible to analyze duplications whose behavior was uncomplicated by association with plasmids, transposons, or integrons. We worked with *P. aeruginosa* because of its complex biology and importance as a pathogen, its exceptional capacity for evolving antibiotic resistance, and because little work on copy number variation has been done with the bacterium (19).

### Duplication construction

**Fig 1:**
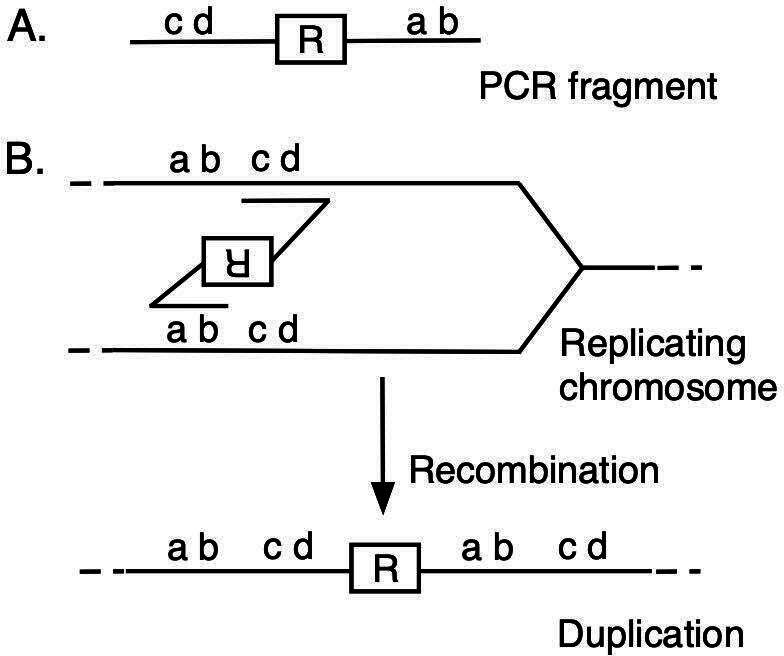
Construction of tandem gene duplications. (**A**) PCR fragment with a selectable marker (“R”) at the novel junction. The fragments typically carry 75 base genome homologies at the ends. (**B**) Recombination with a replicating chromosome creates a gene duplication which carries the selectable marker R (usually for gentamicin resistance).

The procedure we used to create defined duplications in *P. aeruginosa* employs transformation of PCR fragments with genomic homologies in an inverted order relative to the genome and a resistance marker between them at the novel junctions (Fig. 1A) (Materials and Methods) (18). Transformants incorporating the fragments by recombination carry the corresponding duplications and can be selected due to their resistance (Fig. 1B).

To facilitate the recombination events needed to generate duplications, the parent strain carried a plasmid (pNB1) expressing the phage lambda *red* recombinase from an inducible arabinose promoter (20). When the recombinase was induced, PCR fragments with as little as 75 bp homologies at their ends generated recoverable duplications. The short homologies made it possible to create the fragments in a single PCR step using commercial primers, although the recovery of recombinants increased with longer stretches of homology (not shown). The *P. aeruginosa* strain used as a parent for most of this work (“PAO *hsd*”) is a wild-type (*mexS*+) version of strain PAO1 (20) deleted of a restriction system [∆(PA2734-PA2735)] which presumably acts on unmodified PCR fragments. The deletion increased duplication recovery twofold to threefold. A gentamicin resistance determinant (*aacC1*, “*gen*”) was used as the junction marker for most constructions, although other resistance genes were also used successfully (Table S1). In all cases, plasmid pNB1 was eliminated from strains prior to further studies.

### Duplications in three regions of the genome

**Fig 2:**
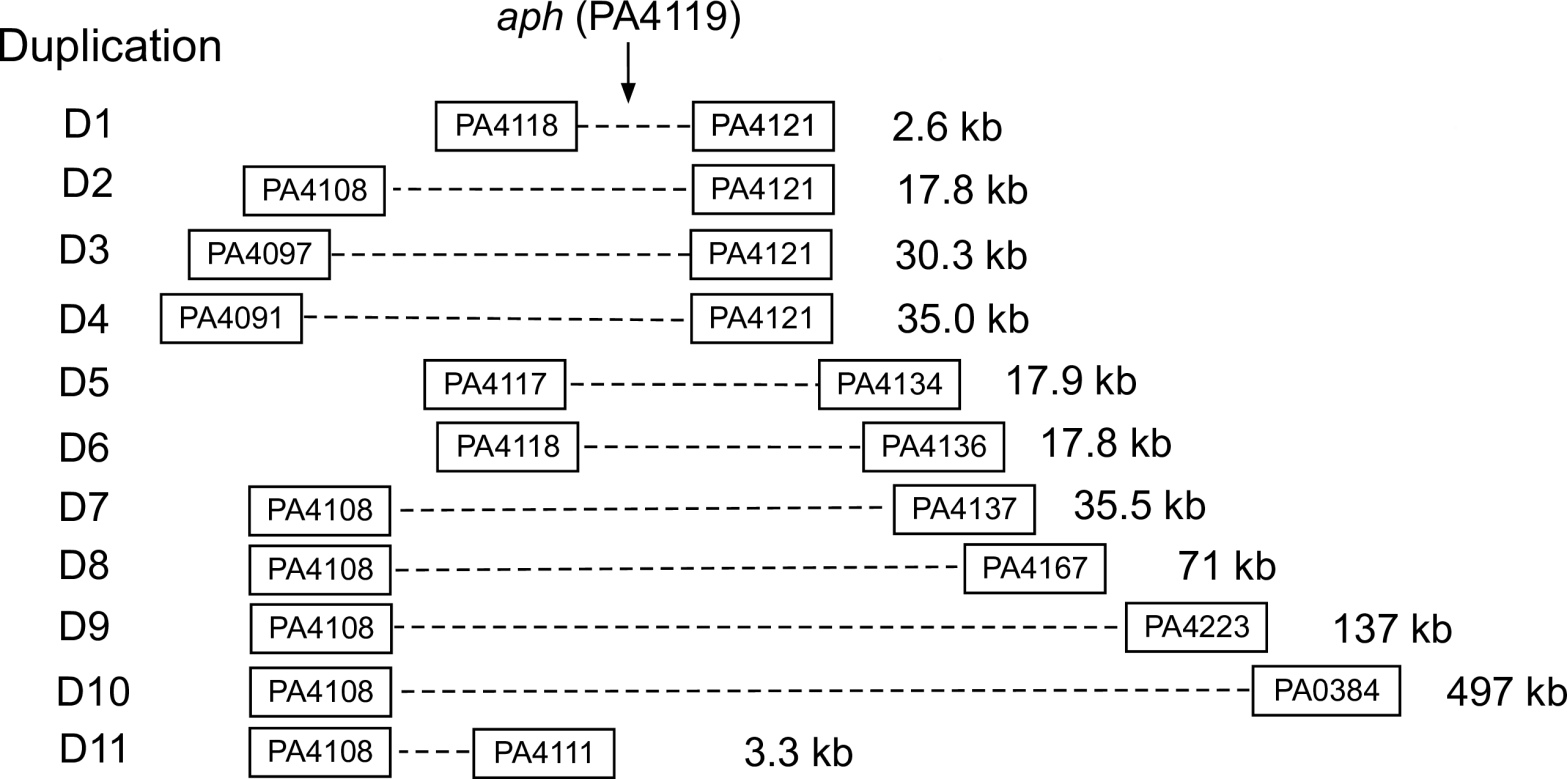
Constructed duplications in the *aph* region. The novel junction marker encodes gentamicin resistance for all 11 duplications. Nine additional duplications in the region are not shown (Table S1). Note that duplication D10 does not include the origin of replication (despite the suggestion it does from the locus numbering) because the parent MPAO1-derived strain carries an *rrnA-rrnB* inversion relative to the PAO1 reference strain (22). Genomic regions are not drawn to scale.

We constructed 31 duplications, 2.6 kb to 497 kb in length, in three separate regions of the genome (Table S1). Duplication structures were verified using traditional PCR amplifications spanning the duplication junctions and droplet digital PCR (ddPCR) assays to quantify copy numbers (Materials and Methods). The largest group of the constructs (20 in total) carries *gen* at their duplication junctions and duplicates genome segments in a region which includes *aph,* a gene encoding an intrinsic kanamycin resistance function that does not act on gentamicin (Fig. 2; Table S1) (21).

The other two genomic regions with duplications included PA2018-PA2019 (*mexXY*) (five duplications) and PA4797 (six duplications) (Table S1). We observed no obvious restrictions on the genomic location or length up to 497 kb in the ability to create duplications using the procedure.

We examined the colony sizes of strains carrying tandem duplications in the *aph* region as a measure of growth fitness. Strains were streaked for colonies and grown overnight on LB agar with or without gentamicin and imaged (Table S2; Fig. S1). Of those tested, only the 497 kb duplication (D10) significantly reduced colony size. Colonies formed by the strain on agar without antibiotic contained many cells which had lost the duplication and grew normally, indicating that the duplication is genetically unstable, as well as slowing growth (see below). The 497 kb duplication also increased the generation time relative to the control and shorter duplications in broth growth (Table S2 legend). We have not investigated the source of the growth defect associated with the 497 kb duplication but note that it is not due to duplication of the *oriT* replication origin (which is not included in the duplication due to a large genome inversion) (22). Although colony size is a sensitive measure of growth rate, we recognize that modest fitness costs may not be evident using this criterion (5).

### Duplication loss is highly correlated with length

**Fig 3:**
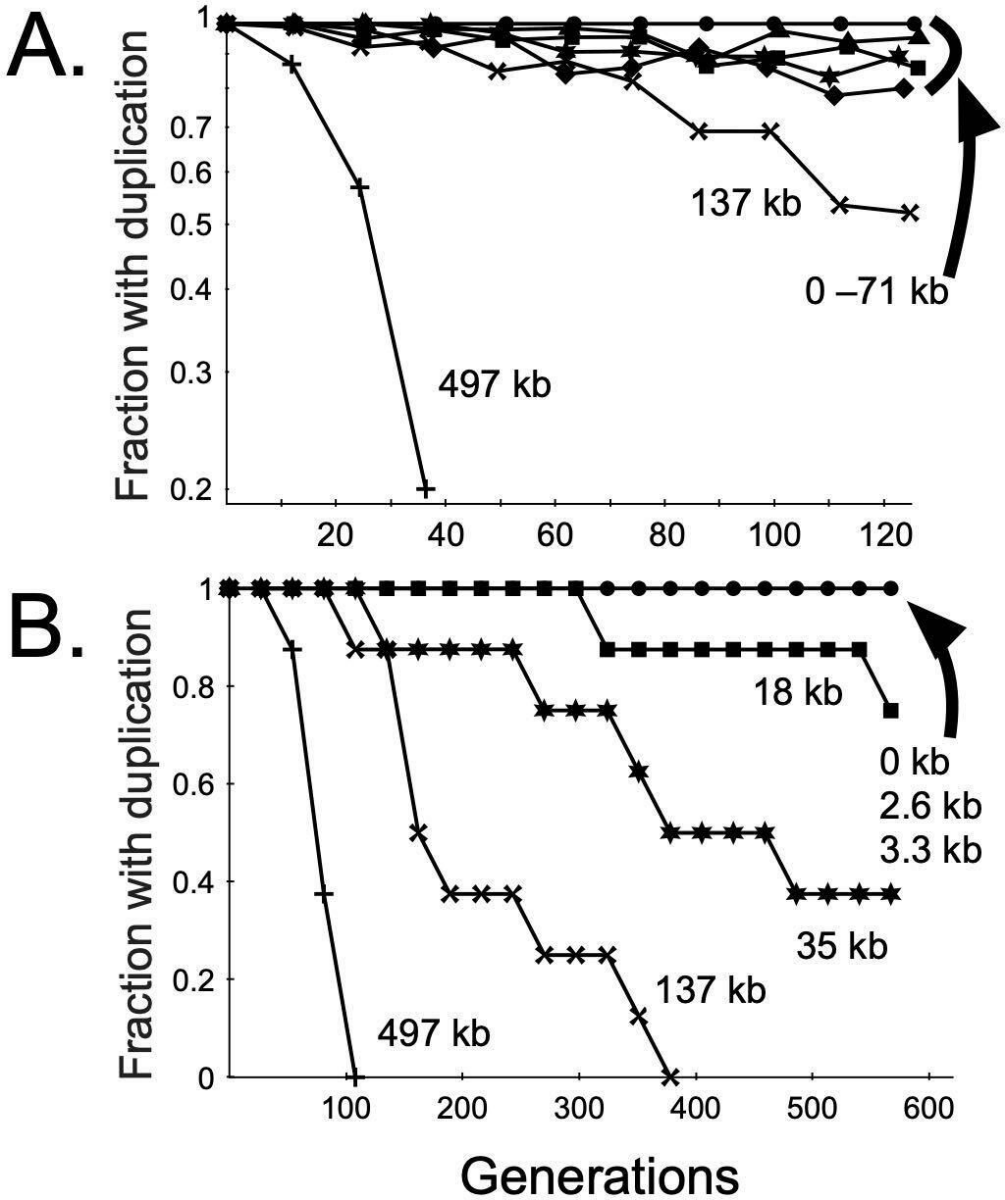
Instability of gene duplications. (**A**) Liquid culture passaging. Strains with the indicated duplications were grown in LB without antibiotic with passaging (1/5,000 dilution into fresh LB) every ~13 generations. The bacteria grow primarily in the exponential phase under these conditions. Data points correspond to geometric means of viable counts (2–3 values) from replicate time courses for all strains except the simple insertion control (“0 kb”) and the 71 kb duplication (geometric standard deviations for replicate values were <1.1 except for the 137 kb 99 [=1.3], 112 [=1.2] generation, and 497 kb duplication 36 generation time points [=2.6]). (**B**) Single colony passaging. For each duplication strain, eight colonies originating from LB-gentamicin agar were streaked daily (~27 generations/day) for 21 days on LB agar lacking antibiotic, taking colonies from each previous streak. Duplication loss (gentamicin sensitivity) was screened for daily. The simple *gen* insertion control for both passaging experiments was strain LP33. After plating for colonies at the end of the experiment, gentamicin-sensitive bacteria could be detected for the 2.6 kb duplication and 3.3 kb duplication strains (1/400 colonies each) but not the simple insertion control (0/400).

Tandem duplications can be genetically unstable and lost or amplified by recombination (Fig. S2). To evaluate the spontaneous loss of duplications we had constructed, we passaged strains carrying them in broth cultures without selection for the gentamicin resistance junction marker for 123 generations and monitored loss phenotypically (gentamicin sensitivity) and by ddPCR (using probes for both the junction gene [*gen*] and the adjacent chromosomal gene [*aph*]) (Fig. 3A). The rate of loss was highly correlated with duplication length. The 497 kb duplication (which reduced fitness) was completely lost by 40 generations, whereas duplications shorter than or equal to 71 kb showed little loss at 123 generations (≤20%) and a 137 kb duplication was intermediate (Fig. 3A). The loss rates per generation ranged over two orders of magnitude, with loss of the two shortest duplications (2.6 and 3.3 kb) being barely detectable (Table 1). The population half-lives of the two short duplications were estimated to be in the thousands of generations, corresponding to months of exponential growth.

**TABLE 1:** Spontaneous loss rates of tandem duplications^*a*^

Strain	Duplication	Length (kb)	Loss rate per generation			50% loss (generations)		
			Gen^R^	*gen*	*aph*	Gen^R^	*gen*	*aph*
LP33	None		<2.5E−05	NA	NA	>28,000	NA	NA
LP40	D1	2.6	~9.8E−05	ND	ND	~7,100	ND	ND
LP50	D11	3.3	~1.6E−04	ND	ND	~4,300	ND	ND
LP41	D2	17.8	9.0E−04	9.0E−04	1.1E−03	770	770	630
LP44	D5	17.9	6.0E−04	ND	ND	1,200	ND	ND
LP43	D4	35	1.2E−03	2.2E−03	2.1E−03	580	320	330
LP46	D7	35.5	1.3E−03	ND	ND	530	ND	ND
LP47	D8	71	2.1E−03	3.0E−03	2.7E−03	330	230	260
LP48	D9	137	5.8E−03	8.5E−03	6.1E−03	120	82	110
LP49	D10	497	7.0E−02	9.8E−02	6.6E−02	10	7	10

^*a*^ The loss kinetics for different duplications during growth with passaging were evaluated by colony patching for loss of the gentamicin resistance phenotype due to *gen* (Gen^R^) and by measuring *gen* and *aph* copy numbers by ddPCR (*gen* and *aph*). Data includes those shown in Fig. 3A and correspond to 2–4 time courses total for each strain. The D1 and D11 values are approximate due to the low rate of duplication loss (i.e., ~1% after 123 generations). Data were fit to exponential decay curves using least squares minimization to estimate 50% loss times and loss rates per generation (decay constants). Duplication loss corresponded to absence of the *gen* junction gene and reduction of *aph* to copy number by 1. Gen^R^, gentamicin resistance; NA, not applicable; ND, not determined.

The stability differences were also seen in colony passaging on agar medium, a procedure akin to that carried out prior to antibiotic sensitivity testing in clinical microbiology laboratories. Eight colonies of different duplication strains were streaked daily (~27 generations/passage) for 21 days on LB agar lacking antibiotic, with duplication loss (gentamicin sensitivity) examined each day. Loss was again highly correlated with length: duplications 35 kb and longer were largely lost by the end of the experiment, whereas duplications 2.6–18 kb (and the simple insertion control) showed little or no loss (Fig. 3B). The persistence of short duplications during growth in liquid and on agar media without selection was unexpected since instability is a hallmark of most tandem duplications.

### Duplications of all sizes amplify readily

We next examined the extent to which a subset of the constructed strains amplified their duplicated segments to higher copy numbers. We assumed that amplification of the *gen* junction marker would confer increased gentamicin resistance (23). We thus selected duplication strain variants exhibiting high-level gentamicin resistance after overnight growth on agar medium and measured copy numbers of their amplified units by ddPCR. We examined both pooled cells from high-density platings and individual resistant colonies (Tables S3 and S4). The assays of the pools provide average copy numbers and showed that all of the duplications exhibited amplifications at one or more gentamicin concentrations, with half reaching averages of 10 or more copies per cell (Table S3). The assays also found that measurements of the junction marker (*gen*) and an adjacent chromosomal gene in the amplified segments (*aph*) gave congruent values, with *aph* present at about one copy more than *gen,* as expected (Table S3 legend). We assume that the amplifications arise from cells with increased segment copy numbers in the initial plated populations and that further amplification may be selected during growth on gentamicin (24).

Three of the duplication strains (4, 7, and 8 in Table S3) showed lower average amplification than the others (fewer than four copies/cell maximum). One of these carries the long 497 kb duplication, which reduces growth fitness markedly, and we assume that high-level amplification exacerbates the growth defect and limits recovery of such derivatives. All three duplications include eight genes (PA4126-PA4133) encoding three membrane proteins, whose overproduction could reduce gentamicin resistance and/or overall growth and thus amplification recovery (20, 25, 26). Indeed, a deletion of the region led to higher average amplification in bacteria carrying the two duplications (strains 10 and 11) but not a control with a duplication that does not include the genes (strain 9) (Table S3).

**Fig 4:**
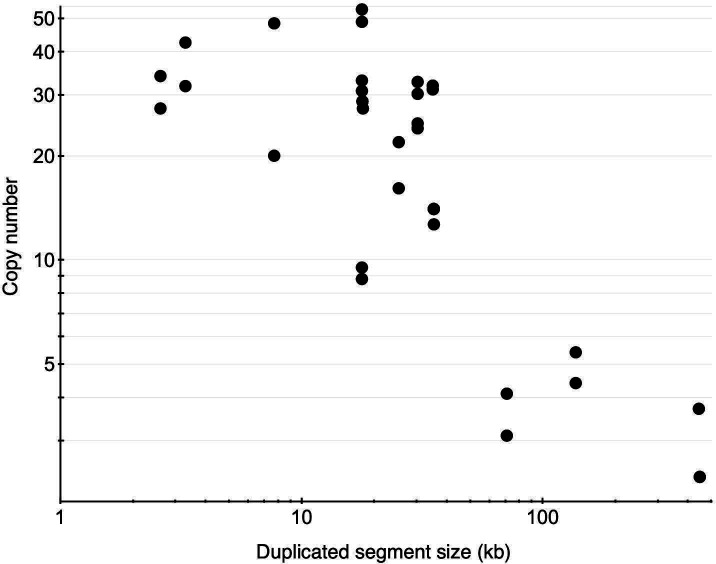
Gene amplifications in individual colonies carrying *gen*-marked duplications. The two highest amplified segment copy numbers observed for each starting duplication are shown (summarized in Table S4). The three longest duplications carry PA4126-PA4133, which could contribute to their apparent limited amplification capacity.

We assumed that the segment copy numbers measured in pooled cells from high-density platings represented averages of cells with different numbers of amplified segments, including those lacking amplifications (and presumably carrying other mutations increasing gentamicin resistance). To test this, we assayed segment copy numbers in 549 resistant individual colonies derived from 15 duplications (Table S4). As expected, colonies carried segment amplifications with different copy numbers. Nearly all of the duplications with segments shorter than 40 kb could amplify markedly (from 9.5 to 53 copies) (Fig. 4; Table S4), whereas longer duplicated segments showed more limited amplification (fewer than six copies). The results show that strains with high segment copy numbers can be recovered from duplication strains after a single cycle of colony formation.

To verify that the copy number values as assayed by ddPCR were accurate, we carried out long-read Oxford Nanopore sequencing on two of the strains with amplifications. For each, the increased read depth of the amplified *aph* regions agreed reasonably well with the corresponding ddPCR values (Fig. S3). The identities of the genome segments amplified were also confirmed to correspond to those expected from the starting duplications, and no additional changes were found in the genome sequences of the amplification strains.

To test whether duplications in a second region of the genome with a different junction marker also amplified, we examined a 16.7 kb duplication of PA4786-PA4805 marked with genes (*aadB* and *aac*) conferring resistance to multiple aminoglycosides. Selections for increased gentamicin resistance were carried out independently four times, and we again saw marked amplification at the higher gentamicin concentrations (up to 29 copies), with relatively reproducible average amplification levels (Table S5). However, we note that the amplified region contained a potential IS element encoded transposase (PA4797) that may have influenced amplification. High copy number amplifications of duplicated segments in two genome regions using different markers could thus be recovered after single steps of colony growth. The result suggests that the potential for high copy number amplification of short duplications is a relatively general feature.

### Diverse mutations cause extreme gentamicin resistance in control strains

Selection experiments with control strains without engineered duplications carrying simple insertions of *gen* (LP33) or *aadB-aac* (LP35) produced small numbers of colonies that were resistant to high levels of gentamicin. We investigated the mechanisms responsible for each marker by analyzing three of each type resistant to 512–1,024 µg/mL gentamicin by ddPCR and whole genome sequencing. We found that none of the *gen* insertions, but all three of the *aadB-aac* insertions, increased the copy number of the corresponding resistant markers by ddPCR (~twofold). Nanopore sequencing found that they carry long duplications of the *rrnC-rrnD* region, which includes the *aadB-aac* marker. Genome sequences of the three *gen* insertion mutants found that one carries a frameshift mutation in *suhB*, a gene associated with aminoglycoside resistance (27). A second carried the *rrnC-rrnD* duplication, which increases resistance without amplifying *gen* (which is located outside the region). A third carried a 34 bp duplication in *rrnB*, a novel rRNA resistance mutation. The fact that five of the six resistance changes are spontaneous tandem duplications is remarkable and helps illustrate the significance of such variants in increasing resistance.

### Intrinsic resistance gene amplification also increases resistance

The studies above involved introduced gentamicin resistance accessory genes as junction markers in duplications. We wished to examine whether amplification of a chromosomal intrinsic resistance gene would also enhance resistance. To do this, we took advantage of the fact that many of our *gen*-marked duplications included *aph* (PA4119), which encodes an unusual metabolic enzyme involved in the utilization of 4-hydroxyphenylacetic acid, which exhibits cross-reactivity with and resistance to kanamycin (28). The *gen* and *aph* products show minimal aminoglycoside cross-reaction (29).

We evaluated the kanamycin resistance of cells carrying multiple *aph* (and *gen*) copies due to amplification (Table S6). We first verified that increased copies of the *gen* gene alone didn’t significantly increase kanamycin resistance by examining strains carrying multiple tandem copies of the *gen* gene but not the *aph* gene derived from duplication D11 (Table S6). Cells carrying as many as 41 copies of the *gen* gene failed to increase the kanamycin MIC more than twofold, as expected (29). In contrast, cells carrying amplifications of *aph* (12–33 copies) in addition to the *gen* gene derived from duplications D1 and D2 increased the kanamycin MIC fourfold to eightfold. The results indicate that increasing the copy number of the *aph* intrinsic resistance gene enhances kanamycin resistance.

### Gene amplifications are intrinsically unstable

**Fig 5:**
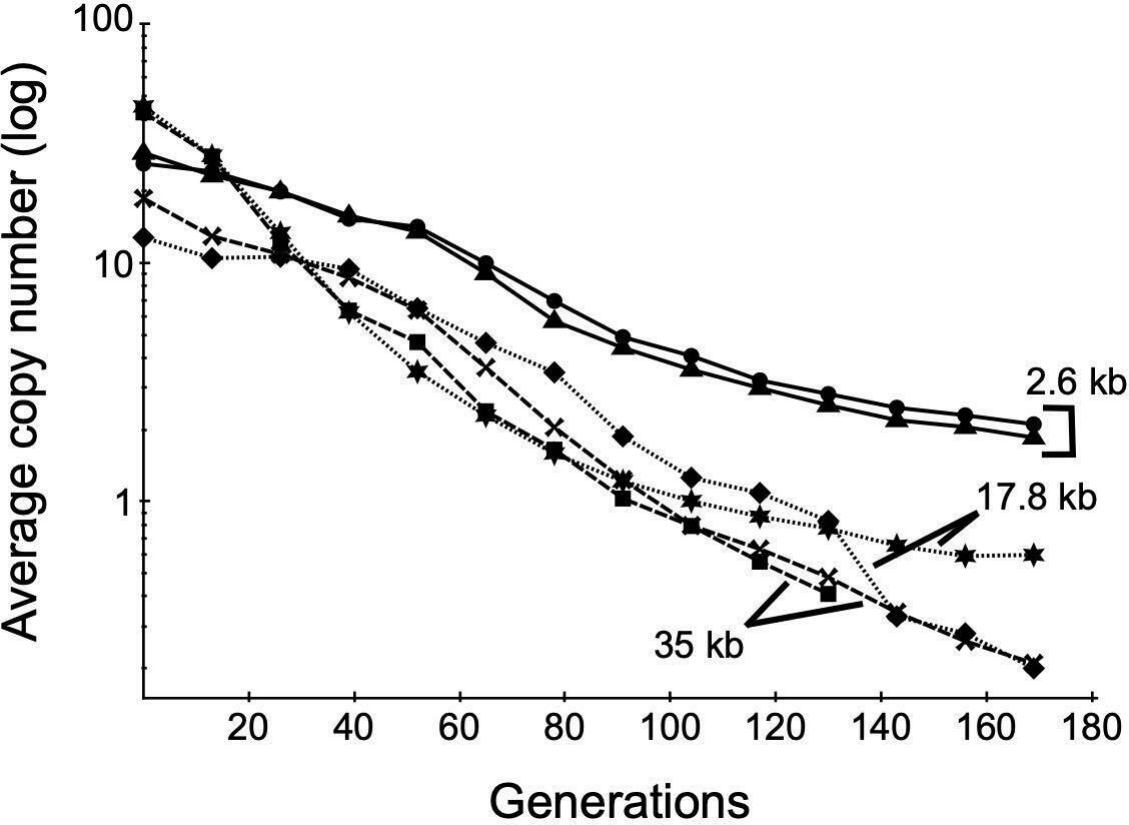
Instability of gene amplifications. The average *gen* junction marker copy numbers of the indicated amplification strains during growth in LB lacking antibiotic with passaging every ~13 generations are shown. Copy numbers were assayed by ddPCR. The amplifications analyzed are derived from D1 (2.6 kb), D2 (17.8 kb), and D4 (35 kb), and biological replicates are indicated by different symbols.

We evaluated the spontaneous reduction in copy number in previously generated amplifications during growth without selection (Fig. 5). There was a rapid reduction of copy numbers for amplifications of 18 and 35 kb units, with nearly complete loss (i.e., average number of junctions <1) for three of four examined after 170 generations. In contrast, amplifications of a 2.6 kb unit were lost more slowly and appeared to plateau at two to three copies. The findings document the overall instability of amplifications of different unit lengths and reinforce the previous finding that short segment duplications tend to persist.

### Extensively passaged duplications retain the capacity for amplification

We sought to examine whether short, genetically stable duplications retained their capacity to amplify and produce highly gentamicin-resistant colonies at elevated frequencies after extended growth without selection. If so, such duplications would represent a potential source of resistance in lineages that had previously developed resistance through gene amplification and later contracted the amplifications to duplication level. We assayed spontaneous resistance for four of the strains (2.6–35 kb) whose duplications had persisted after extended colony passaging without selection (Fig. 3B). We found that all four showed increased frequencies (up to 74-fold) of highly resistant colonies relative to a simple *gen* insertion control, values comparable to those for the corresponding duplication strains that had not been passaged (Table 2). The increased frequencies of resistance in the passaged isolates were again associated with amplification of the duplicated segments in the strains. The results indicate that short duplications, once formed, retain their capacity to amplify and increase resistance after extended growth without selection.

**TABLE 2:** Segment amplification in passaged duplication strains^*b*^

Duplication	Length (kb)	Resistance frequency		Amplification copy number (≥3 units)			
		Unpassaged	Passaged	Unpassaged		Passaged	
				Median	Maximum	Median	Maximum
None (LP33)		5.7E−05	3.1E−05	–^*a*^	–	–^*a*^	–
D1 (LP40)	2.6	1.3E−03	2.3E−03	3.6	4.1	3.5	25
D11 (LP50)	3.3	5.3E−04	1.0E−03	3.3	42.5	3.7	8.3
D2 (LP41)	17.8	1.3E−03	1.1E−03	3.9	6.0	3.7	10
D4 (LP43)	35	4.8E−04	6.6E−04	6.3	6.7	4.2	16

^*a*^ No amplifications observed.

^*b*^ High-level spontaneous gentamicin resistance frequencies and segment amplifications were evaluated for strains carrying the indicated duplication strains before and after ~570 generations growth by passaging on LB agar lacking gentamicin. Efficiency of plating was measured on LB agar with gentamicin (512 µg/mL) vs LB agar alone and corresponds to individual (unpassaged) or geometric means of triplicate (passaged) assays (with geometric SDs 1.2–2.1). The median and maximum copy numbers of amplified units are based on ddPCR analysis of *gen* in single colonies resistant to elevated gentamicin (512 µg/mL) and correspond to those with ≥3 copies of the duplicated units. The numbers of duplication strain colonies showing amplifications for the unpassaged and passaged, respectively, were as follows: none, 0 of 2 and 0 of 25; D1, 2 of 14 and 10 of 25; D11, 5 of 9 and 10 of 25; D2, 8 of 10 and 23 of 25; D4, 3 of 4 and 20 of 25.

## DISCUSSION

In this study, we characterized the genetic behavior of tandem gene duplications of different length in *P. aeruginosa*. In contrast to the transience of long duplications and high copy number amplifications, we found that short duplications exhibited very low rates of spontaneous loss. Such short duplications have not been systematically studied in other species, and their stability may be a general feature. The stability of short duplications suggests that they could regenerate previously experienced adaptive gene amplifications that had been lost.

We examined duplications ranging from 2.6 to 497 kb in length. The duplications were constructed using a PCR fragment-based procedure employing phage lambda *red* recombination. The procedure, analogous to an approach employing natural transformation in *A. baylyi* (18), made it possible to compare duplications of different length from the same genomic region. Most of our duplications carry a gentamicin resistance marker at their junctions to facilitate genetic analysis. It should be possible to extend the procedure developed here to other species for which PCR fragments can be introduced and *red* recombinase is active.

Genetic instability is a hallmark of tandem duplications, and indeed we found that duplications longer than ~100 kb were rapidly lost during growth in the absence of selection. In contrast, short duplications were highly stable. The two shortest duplications analyzed, with duplicated units of 2.6 and 3.3 kb, persisted almost fully in passaging experiments and were estimated to show half-lives in growing populations of thousands of generations. The loss behavior of most of the duplications we analyzed was unlikely to have been greatly affected by fitness costs, since only the longest (497 kb) slowed growth significantly. The stability of the short duplications presumably reflects a combination of limited sequence homology available for recombinational loss and the modest fitness cost of carrying the duplications.

Our findings contrast somewhat with those of a study in *Salmonella enterica* of five long tandem duplications (≥72 kb) showing that the loss rate was only partially correlated with size (30). In this study, the duplications affected different regions of the genome, and their loss could have been influenced by different associated genetic features. A second study of multiple duplications at specific sites in the genome found considerable variation in loss rates, but sequence lengths were not defined (4). The specific loss rates we observed for longer duplications (>70 kb) are comparable to those seen in the earlier studies.

All of the duplications we analyzed could amplify, although short duplications could reach considerably greater unit copy numbers than long duplications, e.g., up to 53-fold for units shorter than 20 kb. This pattern has been seen before (2), and presumably reflects lower fitness costs of highly amplified shorter units compared to longer units (5). Highly amplified segments were lost during growth without selection, as expected (23). However, loss of a short amplified segment (2.6 kb) slowed at low copy number, consistent with higher intrinsic stability of the corresponding duplication.

A possible limitation of this study is that it characterizes constructed duplications rather than those that occur naturally. Naturally occurring duplications are often associated with genetic elements, such as conjugal plasmids, transposons, and integrons, which can influence their behaviors (31–33), and nearly all of our duplications appear free of such nearby elements. However, since most regions of the genome can duplicate spontaneously (1, 4), the constructs we analyzed should be representative of many that occur.

**Fig 6:**
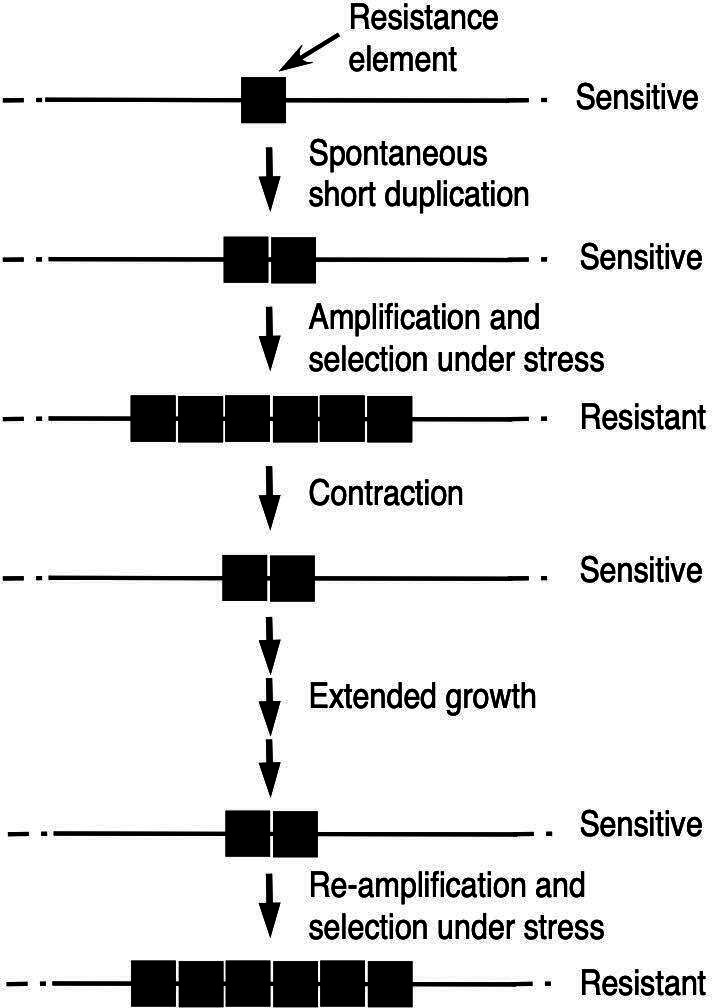
Model for genetic memory conferred by short duplications. The diagram represents a lineage in which a genomic region carrying a resistance gene spontaneously duplicates, followed by amplification and selection of cells carrying the amplification, e.g., due to increased antibiotic resistance. When the selection is removed, cells in which the amplification has contracted to the starting duplication can again take over the population, e.g., due to reduction in fitness cost. The duplication, if short, can persist during extended growth without selection and can re-amplify to provide variants like those originally selected. The model incorporates the relative stability of short tandem duplications into mechanisms established by the groups of Roth, Andersson, Neidle, and others.

The properties of short duplications we documented in this study—stability in the absence of selection and capacity to amplify and de-amplify—suggest that they could provide genetic “memory” of adaptive amplifications selected and then lost (Fig. 6). That is, a spontaneous short duplication (e.g., of a resistance element) could amplify and be selected in response to a stress, such as antibiotic exposure. If the stress ended, a spontaneously de-amplified derivative (e.g., returned to a duplication) could re-emerge and take over the population. Such a derivative should be stable during extended growth without selection but could regenerate the original amplification at an enhanced frequency. Since even the most stable duplications show detectable loss, the genetic memory conferred by short duplications would presumably be shorter-lived than that due to simple mutations like most base pair changes. However, unlike base pair changes, the resistance level could be tunable, in that higher copy numbers could produce greater resistance.

The model can help account for the preponderance of short amplifications among those responsible for antibiotic resistance and heteroresistance (2, 3) and the existence of relatively stable duplications responsible for amplification-mediated heteroresistance (34). The mechanism could be particularly important for bacterial populations adapting to an intermittent stress, such as from “on” and “off” periods of antibiotic exposure in treatment of chronic infections (35). Duplication-mediated genetic memory should, of course, be apropos not only for antibiotic resistance but for any favorable trait conferred by increased copy number of a gene.

## MATERIALS AND METHODS

### Strains

Wild-type strain PAO corresponds to a *mexS*^+^ derivative of strain PAO1 called LPAO (20). PAO *hsd* (LP30) corresponds to LPAO ∆*hsdMS* [∆(PA2734-PA2735)]. Two simple *gen* insertion control strains used were LP33 (LPAO carrying *gen* inserted between PA4108 and PA4109) and LP34 (LPAO ∆PA4121::*gen*), and the simple *aadB-aac* insertion was LP35 [LPAO ∆PA4797::(*aadB-aac*)]. Duplication strains are listed in Table S1. Primers are listed in Database S1.

The ∆(PA2734-PA2735) mutant, which fuses the first seven codons of PA2735 in-frame to the final seven codons of PA2734, was constructed in LPAO using integration-excision (36). A DNA fragment corresponding to the deletion was constructed by extension overlap PCR and introduced into plasmid pU1 (37) by USER cloning (New England Biolabs). The plasmid integrated into the LPAO genome following conjugation from *Escherichia coli* (MFD*pir*) (38), and recombinants that had lost plasmid sequences and recombined the deletion into the chromosome were identified using PCR of individual colonies.

### Duplication construction

Defined tandem duplications were constructed by using PCR fragments (“synthetic bridging fragments”) carrying the duplication novel junctions marked with an antibiotic resistance gene (usually *gen*) for selection (18). Fragments were introduced by electroporation into *P. aeruginosa* expressing phage lambda *red* recombinase (18, 20) and recombined with the genome to generate duplications.

The synthetic bridging PCR fragments were constructed using primers consisting of 5′ 75 bp tails with homology to the target genome regions and 24 bp 3′ sequences homologous to a plasmid-borne gentamicin resistance gene (*gen*) such that the chromosomal homologies flanked the gene in the final PCR products. The 5′ 75-mer primer tails were designed to minimize both self-dimer and heterodimer formation (OligoAnalyzer Tool, IDT, idtdna.com). Approximate target endpoints for each duplication were chosen, avoiding any essential genes (37). For each endpoint location, sequences of 199 possible 75-mer primer sequences that differed by 1 bp attached to the appropriate 5′ or 3′ *gen* sequence (CCCCTGATTCCCTTTGTCAACAGC or GACAATTTACCGAACAACTCCGCG) were examined. Primers showing low hybridization individually (∆G > −10) and with their pair (∆G > −14 in most cases) were purchased (IDT). The *gen* gene was amplified from plasmid pUC18-mini-Tn7T-Gm-lacZ (39) using the 99-mer primers with the Q5U system (NEB), and amplicon DNA was purified (Qiagen QiaQuick PCR purification kit), aiming for a final DNA concentration above 250 ng/µL.

For transformation, *P. aeruginosa* strains with plasmid pNB1, which carries the *red* recombinase gene under arabinose promoter control (20), were grown in LB + carbenicillin (100 µg/mL) overnight at 42°C to OD_600_ ~1.0. Cultures were then diluted to OD_600_ ~0.1 in LB containing carbenicillin (50 µg/mL) + 25 mM MgCl_2_ + 0.3% arabinose and grown with aeration at 37°C to OD_600_ 1.0–1.2 to induce *red* recombinase expression. Cells were made electrocompetent (40) and transformed with synthetic bridging fragment DNA (1 µg), followed by suspension in SOC medium and growth at 37°C with aeration for 3 h and then plating on LB agar containing gentamicin. Following overnight incubation at 37°C, colonies carrying duplications were typically recovered at frequencies of ~10^–7^–10^−8^ per cell.

### MIC determination

Antibiotic MICs were assayed using efficiency of plating spot assays and Etest strips. For the efficiency of plating assays, bacterial colonies were first suspended in LB to OD_600_ 0.15, and 10-fold serial dilutions were spotted onto LB agar containing antibiotic. The resulting plates were grown overnight at 37°C before scoring the MIC, defined as the lowest drug concentration at which the efficiency of plating was less than 5% of that on a control plate lacking antibiotic. For assays using Etest strips (BioMerieux), bacteria were streaked from glycerol freezer stocks to LB agar and grown for 18 h. A population of colonies was suspended in LB to OD_600_ 0.15, then spread onto LB agar. Etest strips were placed on the agar and plates were incubated for 18 h at 37°C before scoring MIC values.

### Droplet digital PCR

Single colonies or cell suspensions were added to Buffer EB (Qiagen) to a final volume of 150 µL in PCR tubes. Cells were boiled at 98°C for 10 min in a thermocycler. A total of 135 µL of boiled cells was added to AFA fiber snap-cap tubes (Covaris 520045). DNA was sheared to 1,500 bp with an M220 focused ultrasonicator (Covaris) using the following shearing parameters: Peak Power = 50, Duty Factor = 2.0, Cycles/burst = 200, Duration = 20 s. Sheared DNA in buffer was removed from the snap-cap tubes and serially diluted 10-fold in water to appropriate levels.

ddPCR probes were designed against one housekeeping gene, PA2830 *htpX* (forward primer: CTTCACCGGCCAGAATTAC, reverse primer: CCATCCACTTGGAGATGAAC, probe: TCTTCTGCGCCGTGTTCGGTTT, HEX fluorophore); target gene PA4119 (*aph*) (forward primer: CCTGTTCGTCAAGCAGGAAG, reverse primer: CTCTGGGTTTCGTTCAGCAC, probe: CTGTCCGCACATGCCGAG, FAM fluorophore); and the gentamicin-resistance marker (forward primer: CATCATTCGCACATGTAGGC, reverse primer: GCTGATGTTGGGAGTAGGTG, probe: CGGCCCTGACCAAGTCAAAT, FAM fluorophore). ddPCR assays were carried out using the Bio-Rad QX200 ddPCR system. DNA was amplified in a C1000 Touch Thermal Cycler utilizing the ddPCR Supermix for probes (no dUTP). Droplet counts were subsequently read in a QX200 Droplet Reader and analyzed for copy number values with QuantaSoft software.

### Duplication loss during growth

Strains to be assayed by broth passaging were streaked from glycerol stocks to LB agar containing gentamicin (30 µg/mL) and incubated overnight at 37°C. Resulting bacteria were suspended in LB broth to a final density of OD_600_ 0.001 (approximately 1:5,000 dilutions to yield ~4.7 × 10^5^ cells/mL) and incubated with aeration at 37°C for 11–12 h (time point 1). For each additional time point, cultures were again diluted to OD_600_ 0.001 and incubated for 11–12 h with aeration at 37°C. At each time point, bacteria were plated for viable counts, and samples frozen or prepared for ddPCR analysis. For some time points, single colonies from platings on LB agar were picked and patched to LB agar containing gentamicin (30 µg/mL) or not to estimate the fractions of gentamicin-sensitive cells in the corresponding cultures. For colony passaging, eight single colonies from different duplication strains grown on LB agar with gentamicin (30 µg/mL) were streaked for colonies on unsupplemented LB agar and grown overnight at 37°C. Resulting individual colonies were again streaked to LB agar and patched to LB agar ± gentamicin (30 µg/mL) to evaluate duplication loss (gentamicin sensitivity) at each time point.

### Oxford nanopore genome sequencing

Cultures of strains to be sequenced were grown overnight from single colonies on LB agar containing gentamicin (30 to 512 µg/mL). Bacteria that grew were inoculated to LB + gentamicin (30 µg/mL) and grown at 37°C with aeration for 4 h to OD_600_ ~2.0. DNA from approximately 1 × 10^9^ cells was isolated following a protocol for isolating high molecular weight DNA using a Nanobind kit (PacBio), and samples were submitted to Plasmidsaurus (plasmidsaurus.com) for sequencing. Reads were aligned to the *Pseudomonas aeruginosa* MPAO1 genome (NCBI assembly accession GCF_016107485.1, ID ASM1610748v1) (https://www.ncbi.nlm.nih.gov/datasets/genome/GCF_016107485.1/) with Minimap2 (41). Alignments were sorted and compressed with SAMTools (42). Gene alignment k-mer counts were tallied with krust (https://github.com/ahcox/krust). Gene k-mer counts were compared to the median count to assess copy number.

### Amplification of tandem duplications

Strains were grown on LB agar + gentamicin (30 µg/mL) for 18 h. Colonies were suspended in LB to an OD_600_ of 0.15. Tenfold serial dilutions were prepped and spotted onto LB agar for pre-selection CFU counts. Tenfold and 100-fold dilutions of the OD_600_ 0.15 suspension were prepped, and 200 µL aliquots were spread on LB + gentamicin agar with two-step concentration increases from 256 to 4,096 µg/mL. A no-antibiotic plating was included. Spread plates were incubated for 18 h at 37°C. Plate contents were harvested by flooding with 2 mL of LB, scraping and mixing the cells with plastic scrapers, pipetting all contents to sterile 15 mL Falcon tubes, and returning any lost volume to 2 mL with additional LB. For ddPCR analysis, 10 µL of cell suspensions were added to 140 µL Buffer EB (Qiagen), and ddPCR prep and analysis was performed as described. Additionally, cell suspensions were 10-fold serially diluted and spotted onto LB agar for post-selection CFU counts.

## SUPPLEMENTAL MATERIAL

Database S1 mbio.02011-25-s0001.xlsx DNA primers.

Supplemental material mbio.02011-25-s0002.pdf Supplemental figures and tables.

ASM does not own the copyrights to Supplemental Material that may be linked to, or accessed through, an article. The authors have granted ASM a non-exclusive, world-wide license to publish the Supplemental Material files. Please contact the corresponding author directly for reuse.
