# Supplementary material for: Short tandem gene duplications as potential agents of genetic memory
Source: mBio. 2025 Oct 16;16(11):e02011-25. doi: 10.1128/mbio.02011-25 (PMC12607827; doi:10.1128/mbio.02011-25)
Supplement: Supplemental material — Supplemental figures and tables. [file mbio.02011-25-s0002.pdf]

Supplemental figures

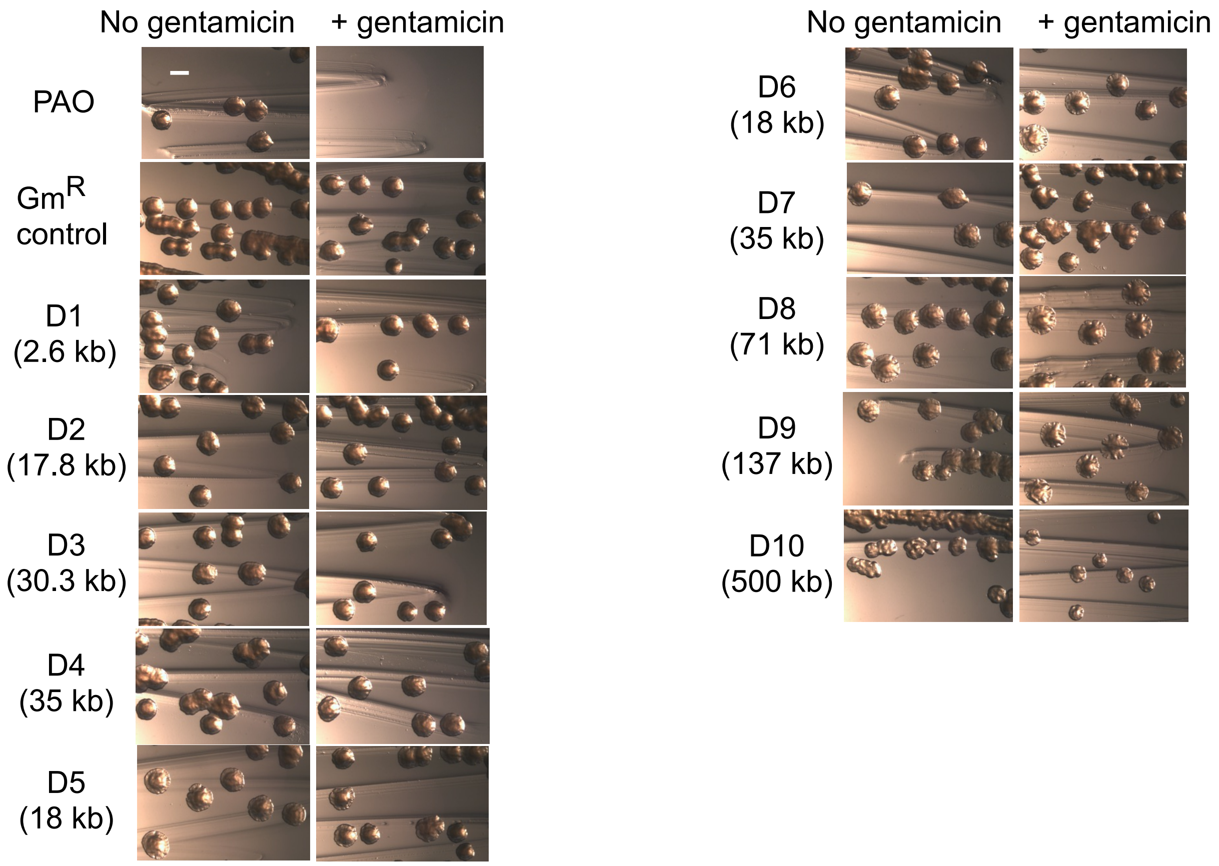

Figure S1. Colonies of strains carrying tandem gene duplications. PAO *hsd* derivatives were grown overnight on LB agar with or without gentamicin (30 µg/ml) as indicated. The gentamicin resistant control corresponds to LP34 (PA4121::*gen)*. Scale bar: 1 mm

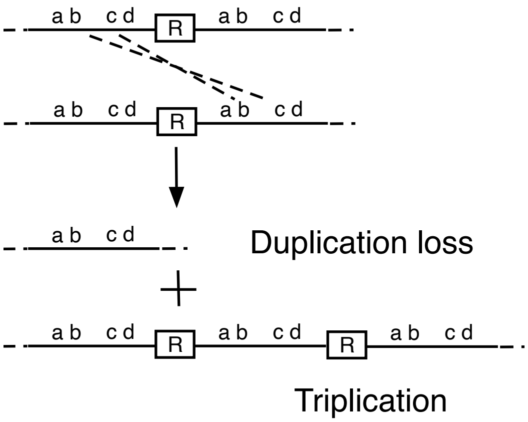

Figure S2. Spontaneous loss and amplification of tandem gene duplications. Unequal reciprocal recombination at duplicated sequences leads to duplication loss and amplification of the duplicated segment to triplications and higher unit copy numbers. Loss can also occur by looping out of one copy of the duplicated segment by intramolecular recombination with failure to replicate.

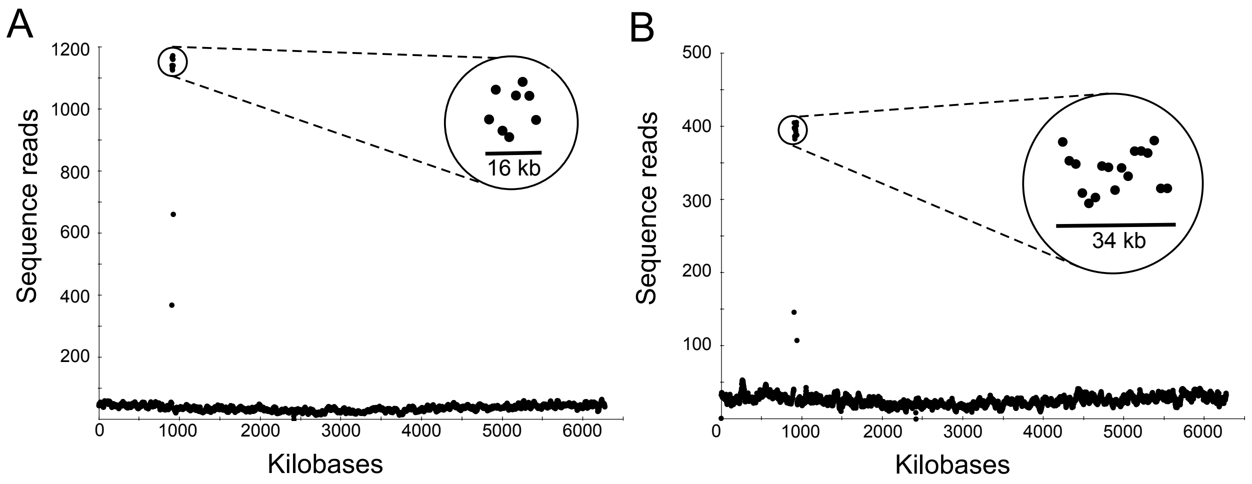

Fig S3. Long-read genome sequence verification of tandem gene amplifications. A., The genomes of cells carrying amplifications of D2 (17.8 kb) and **B.,** D4 (35 kb) were sequenced using Oxford Nanopore technology. Sequence reads per two kb across the genome are plotted, with insets showing the increased reads of the amplified segments. The sequence read/ddPCR copy numbers for the amplified genome segments are 32/38 for D2, and 17/17 for D4.

Table S1. Tandem gene duplications. MIC values were evaluated using agar spot assays, with dilutions of bacteria grown overnight spotted onto LB agar containing antibiotic and incubation for 18 h at 37ºC, with the MIC defined as the lowest antibiotic concentration at which the plating efficiency was less than 5% of the no-antibiotic control plate. ND, not determined; NA, not applicable

**Strain**	**Name**	**Genes duplicated**	**Length (bp)**	**Junction marker**	**Genetic background**	**Gentamicin MIC (µg/ml)**
LP40	D1	PA4118-PA4121	2569	*gen*	PAO *hsd*	256
LP41	D2	PA4108-PA4121	17759	*gen*	"	256
LP42	D3	PA4097-PA4121	30336	*gen*	"	256
LP43	D4	PA4091-PA4121	35050	*gen*	"	256
LP44	D5	PA4117-PA4134	17864	*gen*	"	256
LP45	D6	PA4118-PA4136	17736	*gen*	"	256
LP46	D7	PA4108-PA4137	35502	*gen*	"	256
LP47	D8	PA4108-PA4167	71063	*gen*	"	256
LP48	D9	PA4108-PA4223	137116	*gen*	"	512
LP49	D10	PA4108 -PA0384	496576	*gen*	"	128
LP50	D11	PA4108 -PA4111	3323	*gen*	"	256
LP51	D12	PA4108-PA4121	17759	*gen*	PAO hsd ∆(PA4126-4133)	1024
LP52	D13	PA4097-PA4121	30336	*gen*	"	256
LP53	D14	PA4117-PA4134	7696	*gen*	"	256
LP54	D15	PA4118-PA4136	7568	*gen*	"	256
LP55	D16	PA4108-PA4137	25334	*gen*	"	256
LP56	D17	PA4108-PA4121	17759	*gen*	PAO hsd ∆PA4126	ND
LP57	D18	PA4097-PA4121	30336	*gen*	"	"
LP58	D19	PA4117-PA4134	16586	*gen*	"	"
LP59	D20	PA4118-PA4136	16458	*gen*	"	"
LP60	D21	PA4786-PA4805	16666	*tet*	PAO hsd	NA
LP61	D22	PA4786-PA4805	16666	*aad-aac*	"	"
LP62	D23	PA4786-PA4805	16666	*int-aad-aac*	"	"
LP63	D24	PA4786-PA4805	16666	*tet*	PAO hsd ∆*mexT::gen*	"
LP64	D25	PA4786-PA4805	17197	*tet*	PAO hsd ∆PA4797::*aad/aac*	"
LP65	D26	PA4786-PA4805	17686	*tet*	PAO hsd ∆PA4797::*int/aad/aac*	"
LP66	D27	PA2016-PA2096*	101232	*tet*	PAO	"
LP67	D28	PA2016-PA2096*	100971	*tet*	"	"
LP68	D29	PA2016-PA2043	29233	*tet*	"	"
LP69	D30	PA2016-PA2021	6946	*tet*	"	"
LP70	D31	PA2016-PA2043	29425	*rif*	"	"

* carry different junction regions

Table S2. Colony sizes of duplication strains. Cells were grown into colonies on LB agar lacking gentamicin for 18 h at 37° C. Values were based on measurements of 47-99 isolated colonies for each strain (https://sketchandcalc.com). The control strain lacking a duplication carried a simple *gen* insertion (LP33). We also assayed exponential growth in LB broth with gentamicin (30 µg/ml) for several duplication strains and found the following generation times ±SDs: None, 100±1.6 min; 35 kb, 112±6.4 min; 137 kb, 113±11 min and 497 kb, 132±8.8 min.

Duplication	Length (kb)	Radius (mm)	SD
None	–	0.41	0.07
D1	2.6	0.43	0.07
D2	17.8	0.43	0.07
D4	35	0.44	0.07
D9	137	0.44	0.11
D10	497	0.27	0.11
D11	3.3	0.45	0.07

Table S3. Amplification of tandem duplications. Average genome segment copy numbers of duplicated chromosomal gene *aph* (PA4119) were assayed by ddPCR in pooled colonies harvested after overnight growth on LB agar with or without gentamicin. Average copy numbers of the duplication junction marker (*gen)* were independently assayed for each sample and differed from the corresponding *aph* values in most cases by ~1, as expected, with mean differences ±SEM at different gentamicin concentrations of: 0 µg/ml, 1.02±0.02; 256 µg/ml, 1.09±0.08; 512 µg/ml, 1.84±0.3; 1024 µg/ml, 0.82±0.31; 2048 µg/ml, 0.71±0.24. The approximate number of colony forming units analyzed at each gentamicin concentration were: 0 µg/ml, 3-4 X 10^6; 256 µg/ml, 4-30 X 10^4; 512 µg/ml, 200-2000; 1024 µg/ml, 20-200; 2048 µg/ml, 6-50.

**Strain**	**Duplication (kb)**	**Duplicated loci**	**Genetic background**	**Average genome segment copy number**				
				**Gentamicin concentration (µg/ml)**				
				**0**	**256**	**512**	**1024**	**2048**
1 (None)*	0	None	Wild type	1.0	1.1	1.4	1.1	1.0
2 (D1)	2.6	PA4118-4121	“	2.0	2.2	3.1	5.2	11
3 (D2)	18	PA4108-4121	“	2.1	2.6	5.4	11	4.1
4 (D5)	18	PA4117-4134	“	2.0	2.2	3.9	2.5	2.1
5 (D3)	30	PA4097-4121	“	2.0	2.5	4.8	10	7.8
6 (D4)	35	PA4091-4121	“	2.2	3.0	5.5	9.9	4.3
7 (D7)	36	PA4108-4137	“	2.0	2.3	3.6	2.8	2.6
8 (D10)	497	PA4108-0384	“	1.9	3.2	2.9	2.5	2.4
9 (D13)	30	PA4097-4121	Δ(PA4126-33)	2.1	2.1	5.9	11	5.8
10 (D14)	7.7	PA4117-4134	“	2.1	2.0	11	22	24
11 (D16)	25	PA4108-4137	“	2.1	2.2	7.6	6.3	2.5

* Simple *gen* insertion in PA4121 (strain LP34)

Table S4. Gene amplification in individual highly gentamicin resistant colonies. Individual colonies were assayed by ddPCR for copy numbers of the starting duplicated genome segments. ddPCR assayed the duplicated genome segment marker *aph* (PA4119), or the junction marker *gen* (with copy number values manually increased by 1 to reflect the genome segment copy number). Colonies with amplifications correspond to those with copy numbers ≥3.

Duplication (Strain)	Duplicated loci	Size (kb)	Carries PA4126-33?	Gentamicin (μg/ml)	Colonies with amplifications (≥3 copies)	Amplification copy number	
						Median	Maximum
D1 (LP40)	PA4118–PA4121	2.6	No	30	0 of 3	–	–
				256	1 of 4	3.2	3.2
				512	2 of 14	3.6	4.1
				1024	1 of 8	7.4	7.4
				2048	1 of 15	27.4	27.4
				4096	2 of 10	23.7	34.0
D2 (LP41)	PA4108–PA4121	17.8	No	30	0 of 5	–	–
				256	1 of 10	3.6	3.6
				512	8 of 10	3.9	6.0
				1024	7 of 10	8.6	21.7
				2048	6 of 15	17.5	53.0
				4096	3 of 9	17.4	48.8
D3 (LP42)	PA4097-PA4121	30.3	No	30	0 of 6	–	–
				256	0 of 9	–	–
				512	11 of 11	3.9	5.4
				1024	10 of 10	8.0	23.3
				2048	11 of 13	18.2	30.2
				4096	8 of 9	18.1	32.7
D4 (LP43)	PA4091-PA4121	35	No	30	0 of 5	–	–
				256	0 of 4	–	–

				512	3 of 4	6.3	6.7
				1024	6 of 6	5.2	15.3
				2048	6 of 7	19.4	24.8
				4096	5 of 10	12.8	31.9
D5 (LP44)	PA4117-PA4134	17.9	Yes	30	0 of 4	–	–
				256	6 of 11	4.6	8.4
				512	8 of 11	5.0	8.1
				1024	6 of 10	11.2	28.7
				2048	2 of 14	15.8	28.6
				4096	0 of 8	–	–
D6 (LP45)	PA4118-PA4136	17.8	Yes	30	0 of 2	–	–
				256	0 of 6	–	–
				512	4 of 6	4.2	7.7
				1024	4 of 5	6.5	9.5
				2048	3 of 10	6.7	8.0
				4096	0 of 8	–	–
D7 (LP46)	PA4108-PA4137	35.5	Yes	30	0 of 4	–	2.5
				256	4 of 10	3.6	4.3
				512	1 of 6	5.1	5.1
				1024	6 of 10	11.3	14.4
				2048	0 of 14	–	–
				4096	0 of 8	–	–
D8 (LP47)	PA4108–PA4167	71	Yes	30	0 of 2	–	–
				256	0 of 4	–	–
				512	2 of 4	3.6	4.1
				1024	0 of 2	–	–
				2048	0 of 8	–	–
				4096	0 of 8	–	–

D9 (LP48)	PA4108-PA4223	137	Yes	30	2 of 4	3.6	3.6
				256	3 of 5	3.3	3.7
				512	7 of 8	3.6	4.2
				1024	5 of 7	3.3	4.1
				2048	6 of 7	3.8	5.4
				4096	6 of 8	3.4	4.1
D10 (LP49)	PA4108–PA0384	497	Yes	1024	0 of 2	–	–
				2048	0 of 3	–	–
D11 (LP50)	PA4108–PA4111	3.3	No	512	5 of 9	3.3	42.5
				1024	2 of 10	5.0	5.9
				2048	4 of 10	7.2	13.5
D12 (LP51)	PA4108-PA4121	17.8	No	30	2 of 2	5.7	6.4
				256	6 of 6	6.6	6.8
				512	6 of 6	6.2	6.4
				1024	6 of 6	6.7	9.4
				2048	5 of 5	15.2	33.0
D13 (LP52)	PA4097–PA4121	30.3	No	30	0 of 2	–	–
				256	2 of 6	4.6	4.7
				512	4 of 6	5.4	15.3
				1024	6 of 6	4.9	7.9
				2048	4 of 6	20.7	24.8
D14 (LP53)	PA4117–PA4134	7.7	No	30	0 of 2	–	–
				256	0 of 6	–	–
				512	0 of 6	–	–

				1024	3 of 4	12.5	48.3
				2048	3 of 5	19.5	20.0
D16 (LP55)	PA4108-PA4137	25.3	No	30	0 of 2	–	–
				256	1 of 6	4.6	4.6
				512	6 of 6	4.7	7.1
				1024	4 of 6	11.8	16.1
				2048	1 of 6	21.9	21.9

Table S5. Amplification of an *aadB-aac* marked duplication. Average copy numbers of strain LP61 carrying D22, a 16.7 kb duplication of PA4786-PA4805 with junction aminoglycoside resistance genes *aadB* and *aac* (encoding *ANT(2'')-Ia* and *AAC(6')-Ib9* respectively) were assayed by ddPCR in pooled strains harvested after overnight growth on LB agar containing elevated gentamicin. The experiment was carried out four times independently with bacteria harvested from 10^{-1} or 10^{-2} platings. The viable cell counts (LB agar) in the samples analyzed (median colony forming units per ml± median average deviation) at different gentamicin levels were: 0 µg/ml (1.6E11±4.5E10), 256 µg/ml (1.0E11±3.8E10), 512 µg/ml (2.7E10±2.3E10), 1024 µg/ml (1.1E9±8.7E8), and 2048 µg/ml (9.1E7±8.0E7).

Trial	Average copy number (*aac*) Gentamicin (µg/ml)				
	**0**	**256**	**512**	**1024**	**2048**
1	1.1	1.6	2.9	15	19
2	1.2	1.8	4.2	17	15
3	1.1	1.3	4.6	11	14
4	1.1	1.4	5.8	10	29

Table S6. Enhanced kanamycin resistance associated with amplification of the *aph* gene. The gentamicin and kanamycin resistance levels of bacteria carrying amplification of the *gen* gene alone or both the *gen* and *aph* genes are shown. The *aph* copy number was inferred from the *gen* copy number measured by ddPCR and the structures of the starting duplications. The PAO parent carried ∆*hsdMS* and the *gen* insertion carried *gen* inserted between PA4108 and PA4109.

**Strain**	***aph* copy no.**	***gen* copy no.**	**Kanamycin MIC (µg/ml)**	**Gentamicin MIC (µg/ml)**
PAO	1	0	128	2
gen insertion	1	1	128	256
D11 duplication	1	1	128	256
D11 amplification #1	1	32	128	>4096
D11 amplification #2	1	41	128	>4096
D1 duplication	2	1	128	256
D1 amplification #1	30	29	512	>4096
D1 amplification #2	33	32	512	>4096
D2 duplication	2	1	128	256
D2 amplification #1	14	13	1024	2048
D2 amplification #2	12	11	1024	>4096
